# IT-141, a Polymer Micelle Encapsulating SN-38, Induces Tumor Regression in Multiple Colorectal Cancer Models

**DOI:** 10.1155/2011/869027

**Published:** 2011-12-06

**Authors:** Adam Carie, Jonathan Rios-Doria, Tara Costich, Brian Burke, Richard Slama, Habib Skaff, Kevin Sill

**Affiliations:** Intezyne, Inc, 3720 Spectrum Boulevard Suite 104, Tampa, FL 33612, USA

## Abstract

Polymer micelles are promising drug delivery vehicles for the delivery of anticancer agents to tumors. Often, anticancer drugs display potent cytotoxic effects towards cancer cells but are too hydrophobic to be administered in the clinic as a free drug. To address this problem, a polymer micelle was designed using a triblock copolymer (ITP-101) that enables hydrophobic drugs to be encapsulated. An SN-38 encapsulated micelle, IT-141, was prepared that exhibited potent in vitro cytotoxicity against a wide array of cancer cell lines. In a mouse model, pharmacokinetic analysis revealed that IT-141 had a much longer circulation time, plasma exposure, and tumor exposure compared to irinotecan. IT-141 was also superior to irinotecan in terms of antitumor activity, exhibiting greater tumor inhibition in HT-29 and HCT116 colorectal cancer xenograft models at half the dose of irinotecan. The antitumor effect of IT-141 was dose-dependent and caused complete growth inhibition and tumor regression at well-tolerated doses. Varying the specific concentration of SN-38 within the IT-141 micelle had no detectible effect on this antitumor activity, indicating no differences in activity between different IT-141 formulations. In summary, IT-141 is a potent micelle-based chemotherapy that holds promise for the treatment of colorectal cancer.

## 1. Introduction

It was estimated that there were 1,500,000 new cancer cases and approximately 560,000 deaths out of cancer in 2009 [1]. Chemotherapy is an important treatment option for patients with cancer, however chemotherapy drugs suffer from numerous problems including nonspecific uptake by healthy tissue, poor circulation times, and suboptimal accumulation in the tumor. Often, a large percentage of cytotoxic drug administered to the patient does not reach the tumor environment, but rather is distributed throughout the body, resulting in the many toxic effects associated with chemotherapy and a narrowing of the drug's therapeutic window. The delivery of chemotherapeutic drugs to tumors is still a major hurdle in the eradication of cancer, and the continual development of drug delivery technologies is vital to future breakthroughs in chemotherapy. Polymer micelles offer a promising approach to achieving these goals due to their inherent ability to overcome multiple biological barriers, such as avoidance of the reticuloendothelial system (RES) [2]. Due to their unique size range (20–150 nm), micelles are able to avoid renal clearance (typically less than 20 nm) and uptake by the liver and spleen (particles greater than 150 nm). These micelles can also preferentially accumulate in solid tumors via the enhanced permeation and retention (EPR) effect [3, 4]. The EPR effect is a consequence of the disorganized nature of the tumor vasculature, which results in increased permeability of polymer therapeutics and drug retention at the tumor site. Due to these promising aspects, a number of groups have developed various polymer micelle motifs, encapsulating a wide range of therapeutic classes [5–17].

Colon cancer is the third most common cancer in men and women in most of the developed world [1]. Irinotecan, a topoisomerase I inhibitor, is approved in the clinic for colorectal cancer first-line therapy in combination with 5-fluorouracil/leucovorin/oxaliplatin (FOLFOX) regimen or for monotherapy in second-line therapy following a failed FOLFOX regimen [18]. SN-38, the active metabolite of irinotecan, is about 500–1000 times more cytotoxic than irinotecan [18–20]. Although irinotecan has demonstrated clinical utility, it is highly inefficient in delivering active SN-38 to tumor tissue. Studies in humans have shown that only three to four percent of the administered irinotecan is actually converted to SN-38, which is reliant upon activating carboxylesterase enzymes localized in the liver and gastrointestinal tract [21]. In addition, up to 95% of SN-38 is bound to circulating proteins such as albumin, which drastically reduces its bioavailability [22]. Irinotecan treatment also is accompanied by dose-limiting toxicities of grade 3 and 4 diarrhea and neutropenia [23]. These limitations of irinotecan result in poor exposure of SN-38 to the tumor environment and severe side effects in the patient.

Because of its potency, SN-38 is an attractive molecule for anticancer drug development. A major limitation, however, of free SN-38 is that it is hydrophobic and is unable to be used as a free drug in the clinic. Several groups have addressed the solubility problem of SN-38 by covalently attaching SN-38 to a polymer or peptide [24–26]. In particular, a polymeric micellar formulation of SN-38 based on PEO-poly (glutamic acid) block copolymers through chemical conjugation of SN-38 to the free carboxyl groups present on the poly (glutamic acid) backbone has been developed [26]. This formulation, known as NK012, as well as a peglyated SN-38 formulation (EZN-2208), is currently in clinical trials [27, 28]. While polymer-drug conjugates effectively address solubility of hydrophobic drugs, this prodrug approach is dependent on enzymatic or chemical cleavage of the bond to release the active drug. To develop an encapsulated formulation of SN-38, SN-38 was loaded into a polymer micelle, resulting in aqueous solubility of SN-38 without modification of the drug. This polymer micelle (termed IT-141) was evaluated for pharmacokinetics and antitumor activity compared to irinotecan. The data reported herein support IT-141 as a promising new antineoplastic agent for the treatment of colorectal cancer.

## 2. Materials and Methods

### 2.1. ITP-101 Synthesis

Azido-Poly(ethylene glycol)-t-butyl carbonate-amine (N_3_-PEG-NH-BOC) was prepared as described previously [29]. N-carboxy anhydrides (NCAs) were prepared according to previously published procedures [30, 31]. N_3_-PEG12k-NH-Boc (150 g, 12.5 mmol) was dissolved into 1 L of CH_2_Cl_2_/difluoracetic acid (DFA) (70/30) and was allowed to stir at room temperature overnight. The product was precipitated twice in diethyl ether and was recovered as a white powder (Yield *∼*90%): ^1^H NMR (d_6_-DMSO) 7.77 (3H), 5.97 (1H), 3.83–3.21 (1050 H), 2.98 (2H) ppm.

N_3_-PEG10k-NH_3_/DFA (95 g, 7.92 mmol) was weighed into an oven-dried, 2 L-round-bottom flask and was left under vacuum for three hours before adding the NCA. Asp(OBu) NCA (17.04 g, 79.2 mmol) was added to the flask; the flask was evacuated under reduced pressure, and subsequently backfilled with nitrogen gas. Dry N-methylpyrrolidone (NMP) (560 mL) was introduced by cannula, and the solution was heated to 60°C. The reaction mixture was allowed to stir for 24 hours at 60°C under nitrogen gas. Then, D-Leu NCA (24.88 g, 158 mmol) and Tyr (OBzl) NCA (47.08 g, 158 mmol) were dissolved under nitrogen gas into 360 mL of NMP into an oven-dried, round bottom flask, and the mixture was subsequently added to the polymerization reaction via a syringe. The solution was allowed to stir at 60°C for another three days at which point the reaction was complete (as determined by HPLC). The solution was cooled to room temperature, and diisopropylethylamine (DIPEA) (10 mL), dimethylaminopyridine (DMAP) (100 mg), and acetic anhydride (10 mL) were added. Stirring was continued for 1 hour at room temperature. The polymer was precipitated into diethyl ether (10 L) and isolated by filtration. The solid was redissolved in dichloromethane (500 mL) and precipitated into diethyl ether (10 L). The product was isolated by filtration and dried *in vacuo* to give the block copolymer as an off-white powder (134.6 g, Yield = 73%): ^1^H NMR (d_6_-DMSO) *δ* 8.43–7.62 (50H), 7.35 (100H), 7.1 (40H), 6.82 (40H), 4.96 (40H), 4.63–3.99 (50H), 3.74–3.2 (1500H), 3.06–2.6 (60H), 1.36 (90H), 1.27–0.47 (180).

N_3_-PEG12 K-*b*-Poly(Asp(OBu)_10_)-*b*-Poly(Tyr(OBzl)_20_-*co*-D-Leu_20_)-Ac (134.6 g, 6.4 mmol) was dissolved into 1000 mL of a solution of pentamethylbenzene (PMB, 0.5 M) in trifluoroacetic acid (TFA). The reaction was allowed to stir for five hours at room temperature. The solution was precipitated into a 10-fold excess of diethyl ether, and the solid was recovered by filtration. The polymer was redissolved into 800 mL of dichloromethane and precipitated into diethyl ether. An off-white polymer was obtained after drying the product overnight *in vacuo* (111.8 g, Yield = 93%): ^1^H NMR (d_6_-DMSO) *δ* 12.2 (10H), 9.1 (10H), 8.51–7.71 (50H), 6.96 (40H), 6.59 (40H), 4.69–3.96 (60H), 3.81–3.25 (1500H), 3.06–2.65 (60H), 1.0–0.43 (180). ^1^H NMR (d_6_-DMSO) *δ* 171.9, 171, 170.5, 170.3, 155.9, 130.6, 129.6, 127.9 115.3, 114.3, 70.7, 69.8, 54.5, 51.5, 50, 49.8, 49.4, 36.9, 36, 24.3, 23.3, 22.3, 21.2. IR (ATR) 3290, 2882, 1733, 1658, 1342, 1102, 962 cm^−1^.

### 2.2. IT-141 Formulation

SN-38-loaded micelles were prepared by dissolving 1 g of ITP-101 in 200 mL of water and 100 mg of SN-38 in 8 mL of methanol and 16 mL of toluene. The water was mixed with a Silverson LT4R shear mixer at 10,000 rpm at 4°C, and the organic solution was added dropwise. The solution was mixed for 30 minutes, then the resulting emulsion gently stirred on a magnetic stir plate overnight, allowing the toluene to evaporate. The SN-38-loaded micelle solution was filtered through a 0.22 *μ*m PES filter, then lyophilized to give a slightly yellow powder.

### 2.3. High-Performance Liquid Chromatography

The HPLC instrumentation consisted of a Waters Alliance separation module (W2695) equipped with a Lichrosphere Select B (5 *μ*m), 250 × 4.6 mm column coupled with a Waters multi-wavelength fluorescence detector (W2475) with excitation at 355 nm and emission at 515 nm. Mobile phase consisted of a 70 : 30 phosphate buffer (10 mM NaH_2_PO_4_, 0.1% TEA, pH 3.5)/acetonitrile. Flow rate was isocratic at 0.8 mL/min. Elution time for SN-38 was determined to be 11.6 minutes, while camptothecin internal standard was 4.2 minutes.

### 2.4. Size and Zeta Analysis of IT-141

Particle sizes were determined using dynamic light scattering on a Wyatt DynaPro (Santa Barbara, Calif). Micelle solutions were prepared at 1 mg/mL in filtered water and were centrifuged at 2,000 rpm to remove any dust prior to analysis. Zeta measurements were performed on a Malvern Zetasizer (Worcestershire, United Kingdom).

### 2.5. Drugs, Cell Lines, and Animals

SN-38 was purchased from Yingxuan Pharmaceuticals (Shanghai, China). Camptothecin and irinotecan were purchased from Sigma. All cells were purchased from American Type Tissue Collection (ATCC) and maintained in the following media: RPMI 1640 with 10% FBS, 2 mM L-Glutamine, and 100 units/mL penicillin/streptomycin (LNCaP, PC-3, MG-63, BxPC-3, MCF-7, and BT-474), DMEM with 10% FBS, 2 mM L-Glutamine and 100 units/mL penicillin/streptomycin (MDA-MB-453, MDA-MB-231), F12K with 10% FBS, 2 mM L-Glutamine and 100 units/mL penicillin/streptomycin (A549), and McCoy's 5A with 10% FBS, 2 mM L-Glutamine, and 100 units/mL penicillin/streptomycin (HT-29 and HCT116). All media, FBS, and supplements were purchased from Mediatech (Manassas, Va) or Hyclone. Female athymic nude mice weighing about 20–25 g were obtained from Charles River Laboratories (Wilmington, Mass).

### 2.6. Cytotoxicity Assay

For assessing cytotoxicity, cancer cell lines were plated in 96-well white-walled plates. The following day, when the cells were 50% confluent, the cells were treated with IT-141, free SN-38, or irinotecan in complete growth medium. IT-141 was administered using SN-38-equivalent concentrations based on the weight loading of the formulation. The drugs remained on the cells for 72 hours without media change. At this timepoint, cell viability was determined using the Cell Titer Glo kit and measured using a luminescent plate reader (BMG Labtech, Cary, NC). Cells were treated in triplicate. Data are presented as mean ± standard deviation.

### 2.7. Pharmacokinetic Studies

HT-29 cells were subcutaneously injected into the right flank of nude mice at a concentration of 5 million in 0.1 mL PBS. When the tumors were approximately 300 mm^3^, mice were randomly divided into two groups of eight and injected with 30 mg/kg (SN-38-equivalent) of IT-141 or 30 mg/kg irinotecan. Injection occurred by a fast IV bolus into the tail vein in a volume of 0.2 mL. The delivery vehicle for IT-141 was isotonic saline and acidified (pH 3.5) isotonic saline for irinotecan. Mouse blood was collected at timepoints of 5 minutes, 15 minutes, 1 hour, 4 hours, 12 hours, 24 hours, and 72 hours. Tumors were excised at the same timepoints, and snap frozen. plasma was isolated by centrifugation at 2000 rpm for 5 minutes. Plasma was processed for HPLC analysis by protein precipitation in ice-cold, acidified methanol (10% perchloric acid/methanol) with 100 ng/mL camptothecin as internal standard, at a ratio of 1 : 4 plasma to methanol. Tumors were homogenized in 20 mM ammonium acetate, pH 3.5 and extracted in acidified methanol as described above. Samples were vortexed for 10 minutes, centrifuged at 13,000 rpm for 10 minutes, and the supernatant was transferred to HPLC vials for analysis. Data are presented as mean ± standard deviation.

### 2.8. Maximum Tolerated Dose (MTD) Studies

HT-29 cells were subcutaneously injected into the right flank of nude mice at a concentration of 5 million in 0.1 mL PBS. When the tumors were approximately 300 mm^3^, mice were given both single and multidose (Q4D × 3, Day 0, 4, 8) intravenous injections of IT-141 at doses ranging from 10–90 mg/kg. Body weight was recorded every other day. The MTD was defined as a dose that caused no greater than a 10% loss in body weight and no treatment-related deaths.

### 2.9. Antitumor Efficacy Studies

HT-29 and HCT-116 colon cancer cells were harvested and resuspended in sterile PBS at a concentration of 2 million (HT-29) or 4 million (HCT-116) cells per 0.1 mL PBS and injected subcutaneously into the right flank of athymic nude mice. Tumors were allowed to establish logarithmic growth (*∼*7–14 days), and the animals were randomly divided into six to eight mice per group. Drug was administered by a fast bolus injection of 0.2 mL into the mouse tail vein on a schedule of Q4D × 3. Bidimensional tumor measurements were made with calipers once every other day. Tumor volume was calculated according to the formula: *V* = (short diameter)^2^(long diameter)/2. Percent inhibition was calculated using the following formula:

(1) $$100-\frac{V_{\text{group}}-V_{\text{group}\;0}}{V_{\text{Ctl}}-V_{\text{Ctl}\;0}}\times 100,$$

where *V*_group_ is the tumor volume on the final day of the study, *V*_group  0_ is the tumor volume of the group on day 0, *V*_Ctl_ is the tumor volume of the control group on the final day of the study, and *V*_Ctl  0_ is the tumor volume of the control group on day 0. Tumor regression was calculated using the following formula:

(2) $$\;\frac{V_{\text{group}\;0}-V_{\text{group}}}{100}\times 100,$$

where *V*_group_ is the tumor volume on the final day of the study and *V*_group  0_ is the tumor volume of the group on day 0. Statistical differences in tumor volume between groups were calculated using the Student's *t*-test using Microsoft Excel, whereby *P* < 0.05 was considered statistically significant. Data are presented as mean tumor volume ± standard error.

## 3. Results

**Figure 1:**
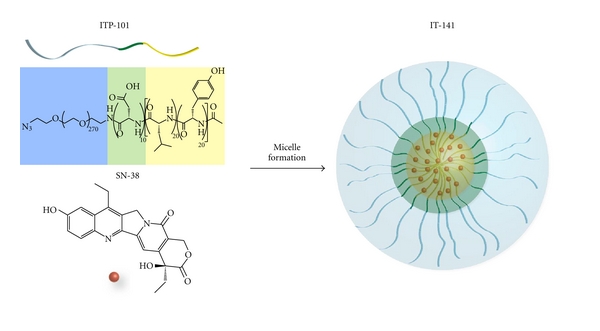
Diagram of the IT-141 formulation. ITP-101 consists of poly(ethylene glycol)-*b*-poly(aspartic acid)-*b*-poly(D-leucine-*co*-tyrosine), where the hydrophobic amino acids provide a core region into which a hydrophobic drug can reside, and the amphiphilic PEG block forms a protective corona around the micelle. Addition of SN-38 (shown in red) to ITP-101 (see Section 2) forms an SN-38-loaded micelle, termed IT-141.

ITP-101 is a triblock copolymer consisting of poly(ethylene glycol)-*b*-poly(aspartic acid)-*b*-poly(D-leucine-*co*-tyrosine). The hydrophobic amino acids provide a core region into which a hydrophobic drug can reside, and the amphiphilic PEG block forms a protective corona around the micelle, giving the delivery system stealth-like properties to avoid protein opsonization and RES uptake (Figure 1). The use of both D and L stereoisomers of amino acids in the leucine/tyrosine core block disrupts the secondary structure of the polypeptide. Replacing the rodlike helical nature of the polypeptide with the flexibility of a random coil allows for significant increases in drug loading efficiency (data not shown). The middle aspartic acid block allows for a hydrogen-bonding segment which can be further stabilized with the use of metal ions, an aspect that is not utilized for IT-141.

IT-141 was formulated using ITP-101 with various concentrations of SN-38, ranging from 1 to 14% (w/w), achieving greater than 90% loading efficiency. Formulations of IT-141 reconstituted in water or saline resulted in a homogeneous solution free of precipitate for up to four days at room temperature, and the lyophilized powder is stable for months. Following formulation, the aqueous solubility of SN-38 in IT-141 was 30 mg/mL, which is about a 6,000-fold increase in solubility of SN-38. [25]. Dynamic light scattering (DLS) experiments demonstrated that the micelle size was approximately 130 nm, with a standard deviation of ±6 nm. Thus, the average size of IT-141 falls within the desired range to avoid renal clearance (above *∼*20 nm) and escape uptake by the RES (below *∼*150 nm). Zeta potential measurements from electrophoretic light scattering experiments demonstrated that the surface charge of the micelle is overall neutral, with a range of readings from −5 to 5 mV. 

The sensitivity of various cancer cell lines to free SN-38, IT-141, and irinotecan was compared in a cytotoxicity assay. As shown in Table 1, both free SN-38 and IT-141 were extremely potent, and the sensitivity of the cells to IT-141 was similar to free SN-38 across the cell lines. Irinotecan was several orders of magnitude less toxic than either free SN-38 or IT-141. Certain cell lines (PC-3, MDA-MB-231, and BT-474) were insensitive to both free SN-38 and IT-141.

**Figure 2:**
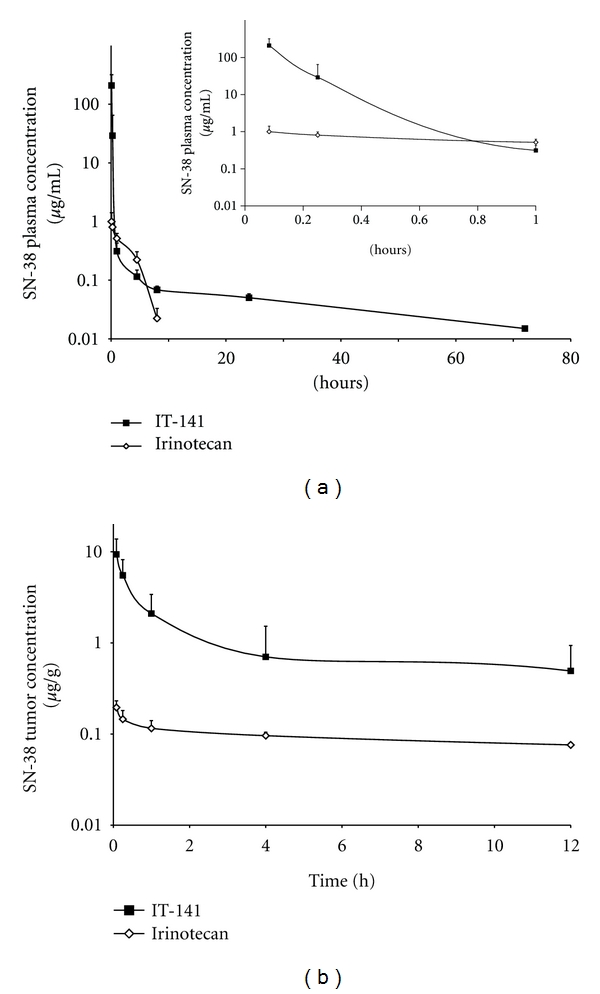
Plasma and tumor pharmacokinetics of IT-141 compared to irinotecan. (a) HT-29 tumor-bearing nude mice (eight mice per group) were administered a single bolus intravenous injection of IT-141 or irinotecan at a dose of 30 mg/kg. (a) Plasma concentration of SN-38 compared to irinotecan plotted versus time. Inset: plasma concentrations plotted from 5 min to 1 hour. (b) Tumor concentration of SN-38 compared to irinotecan plotted versus time. Values are presented as mean ± standard deviation.

To determine the MTD of IT-141, HT-29 tumor-bearing nude mice were given both single and multidose (Q4D × 3) intravenous injections of IT-141. These studies demonstrated that the multidose MTD of IT-141 in tumor-bearing animals was 45 mg/kg and single dose MTD was 60 mg/kg. Using 30 mg/kg of IT-141 as a safe dose, the pharmacokinetic (PK) profile and tumor accumulation of SN-38 delivered from IT-141 then compared to irinotecan in nude mice bearing HT-29 tumors (Table 2). Mice receiving a single injection of 30 mg/kg IT-141 achieved a significant improvement in SN-38 plasma concentration and exposure compared to 30 mg/kg of irinotecan (Figure 2(a), Table 2). The *C*_max⁡_ for both groups was achieved by the first measured time point of 5 minutes, with >200-fold higher SN-38 concentration in mice treated with IT-141 (209 *μ*g/mL) compared to irinotecan (1.0 *μ*g/mL). SN-38 exposure as measured by area under curve (AUC) from irinotecan was 2.5 *μ*g∗hr/mL, while SN-38 exposure from IT-141 was 13.8-fold greater at 34.6 *μ*g∗hr/mL. No data could be obtained for irinotecan plasma concentrations beyond 12 hours as the concentration fell below the limit of detection. The concentration of SN-38 in the tumor over time is plotted in Figure 2(b). The tumor AUC of IT-141 was determined to be 16.4 *μ*g∗h/g, which was significantly higher than irinotecan at 1.9 *μ*g∗h/g. IT-141 also had a 47-fold higher *C*_max⁡_ in the tumor than irinotecan (9.4 *μ*g/mL versus 0.2 *μ*g/mL). 

**Figure 3:**
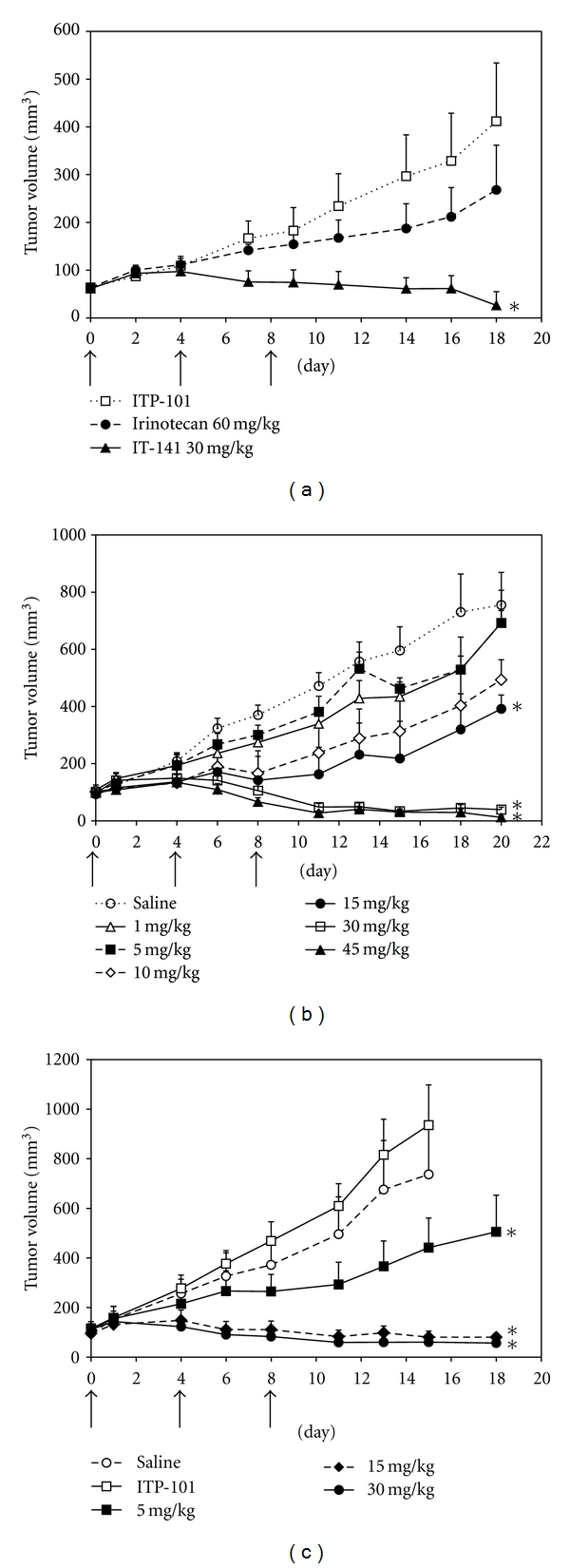
Antitumor efficacy of IT-141 in colorectal cancer xenograft models. (a) HT-29 tumor-bearing mice (eight mice per group) were injected intravenously with ITP-101 alone (300 mg/kg), IT-141 (30 mg/kg), or irinotecan (60 mg/kg) on a Q4D × 3 schedule. (b) HT-29 tumor-bearing mice (seven mice per group) were injected with 1, 5, 10, 15, 30, or 45 mg/kg of IT-141 along with a saline control group. (c) HCT-116 tumor-bearing mice (six mice per group) were injected with 5, 15, or 30 mg/kg of IT-141 along with saline and ITP-101 control. Asterisks denote that treatment group is statistically different from control group (*P* < 0.05). Arrows indicate days of injection (days 0, 4, and 8). Data are presented as mean tumor volume ± standard error.

Based on the pharmacokinetic data, it was hypothesized that IT-141 would show superior antitumor efficacy in colon cancer xenograft models compared to irinotecan. To test the antitumor efficacy of IT-141, HT-29 tumor-bearing mice were treated with either ITP-101 alone at 300 mg/kg, irinotecan at 60 mg/kg, or IT-141 at 30 mg/kg (Figure 3(a)). Treatment with irinotecan at 60 mg/kg, which is near its MTD on this dosing schedule, did not inhibit HT-29 tumor growth significantly compared to polymer alone [26, 32]. However, treatment with IT-141 at half the dose of irinotecan induced significant tumor regression by day 18, ultimately resulting in complete inhibition of tumor growth compared to ITP-101 control and 35% regression from initial tumor volume (*P* = 0.002). Dose-ranging studies were then performed to determine if the antitumor efficacy of IT-141 is dose dependent (Figure 3(b)). HT-29 tumor-bearing mice were intravenously administered IT-141 at doses of 1, 5, 10, 15, 30, and 45 mg/kg via tail vein injection. Treatment with 1, 5, or 10 mg/kg did not result in a statistically significant inhibition of tumor growth compared to control mice receiving only saline. By day 20, treatment with 15 mg/kg IT-141 resulted in a 54% inhibition of tumor growth, respectively, compared to mice treated with saline (*P* = 0.028). Treatment with 30 and 45 mg/kg resulted in complete tumor growth inhibition compared to saline control, with tumor regression of 59 and 87%, respectively (*P* = 0.005 for both). 

Similar results were found using another colon cancer xenograft model, HCT116 (Figure 3(c)). In this model, a dose of 5 mg/kg resulted in a 59% inhibition of tumor growth (*P* = 0.008) compared to the ITP-101-treated group. Treatment with IT-141 at 15 and 30 mg/kg in this model resulted in complete inhibition of tumor growth compared to the ITP-101 polymer control, with 15% and 51% regression, respectively (*P* = 1.0 *e* ^−4^ and 8.1^−5^). Taken together, these data demonstrate that IT-141 achieved significantly greater antitumor efficacy, compared to irinotecan, and dose-dependent tumor regression in two colorectal cancer xenograft models of colon cancer, with effective doses between 15 and 30 mg/kg. 

**Figure 4:**
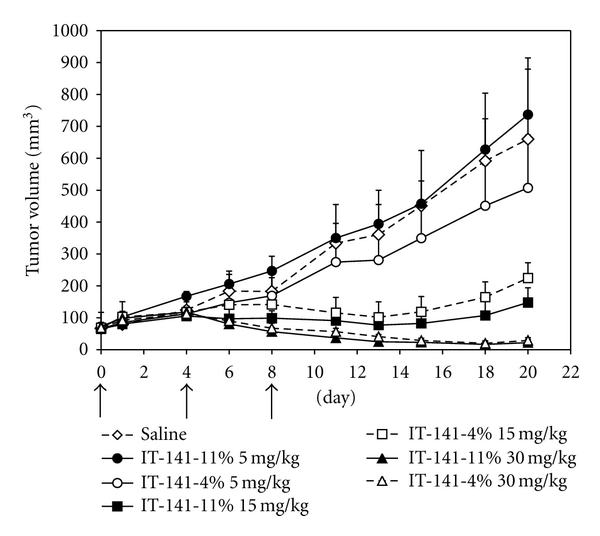
Antitumor efficacy of different weight loadings of IT-141 in an HT-29 xenograft model. IT-141 was formulated to contain either 4 or 11% SN-38 by weight and was administered i.v. to nude mice bearing HT-29 tumors at 5, 15, or 30 mg/kg. Each group contained six mice. Arrows indicate days of injection (days 0, 4, and 8). Data are presented as mean tumor volume ± standard error.

A final study was performed whereby IT-141 formulations with different weight loadings of SN-38 were compared to each other. IT-141 formulations were prepared with 11% (IT-141-11%) and 4% (IT-141-4%) SN-38 (w/w), and equivalent doses of SN-38 were administered i.v. in an HT-29 colon cancer xenograft model (Figure 4). There were no statistical differences in efficacy between the two formulations at either 30 mg/kg (*P* = 0.292), 15 mg/kg (*P* = 0.119), or 5 mg/kg (*P* = 0.138). These data demonstrate that the percent loading by weight of SN-38 into the micelles does not affect antitumor activity.

## 4. Discussion

In this report, a novel triblock copolymer was used to encapsulate and solubilize the hydrophobic drug, SN-38, which is the active metabolite of irinotecan. Although irinotecan is used in the clinic as a prodrug, its efficacy is reliant upon carboxylesterase enzymes localized in the liver and gastrointestinal tract for conversion to the active metabolite, SN-38. Irinotecan treatment is often followed by late-stage diarrhea with 24% grade 4 incidence and can require antidiarrheal premedication [33]. This limits the dose of irinotecan that can be administered safely in subsequent administrations, thereby reducing response rates in these patients [34, 35]. SN-38 is a potent cytotoxic compound that, by itself, cannot be used in the clinic due to its extreme hydrophobicity. Hamaguchi et al. have effectively addressed the solubility problem of SN-38 by conjugating SN-38 to PEG-poly(glutamic acid), forming a micelle called NK012, which is currently in clinical trials [27]. Other nanocarriers for SN-38 have been developed involving conjugation of SN-38 to a polymer or peptide [24, 25]. As an alternative approach to direct SN-38 conjugation, a novel triblock copolymer was used to encapsulate SN-38 into a polymer micelle, precluding the need to modify the drug and for cleavage of the bond to release the active drug. The ITP-101 triblock copolymer was developed to efficiently encapsulate hydrophobic molecules and release them at the site of disease (in the tumor) without drug conjugation.

Encapsulation of SN-38 to create IT-141 resulted in a 6,000-fold increase in solubility of SN-38 and a micelle size of 130 nm, which is ideal for accumulation in tumors due to the EPR effect [36]. In vitro, IT-141 was found to possess potent cytotoxic activity, which was similar to that of free SN-38 but several fold more potent than irinotecan. Cell lines that were resistant to killing by IT-141 were also resistant to free SN-38, which may indicate a natural insensitivity of these cell lines to inhibition of topoisomerase I. This could arise through alterations in the expression of, or mutations in, the gene encoding topoisomerase I or the activity of drug efflux pumps [37]. It has been shown that the drug efflux pump ABCG2 is overexpressed in cells resistant to SN-38 [38].

The pharmacokinetic profile of IT-141 demonstrated significant improvement in exposure and *C*_*Max*⁡_ for SN-38, with a modest improvement in half-life, compared to SN-38 derived from irinotecan. Importantly, the plasma AUC from IT-141 exposure was 14-fold higher than the SN-38 exposure from irinotecan administered at the same dose (34.6 *μ*g∗hr/mL versus 2.5 *μ*g∗hr/mL). Similarly, IT-141 demonstrated higher exposure in HT-29 tumors, as measured by AUC, than irinotecan. The higher AUC of IT-141 in the tumor indicated that it would potentially be more efficacious than irinotecan in xenograft models. Indeed, IT-141 was found to be superior to irinotecan in an HT-29 xenograft model and was potent in dose-range finding studies in both HT-29 and HCT-116 xenografts. In both models, tumor regression was observed at 30 mg/kg in the HT-29 model and 15 mg/kg in the HCT116 model. 

During the development of IT-141, it was found that IT-141 could be formulated with SN-38 with weight loadings in the range of 1–14%. Different IT-141 formulations were prepared with varying weight loadings of SN-38 and were evaluated in an HT-29 xenograft experiment. It was found that IT-141-4% w/w had equivalent antitumor activity to IT-141-11% w/w, demonstrating no differences in efficacy between these formulations. It can be speculated, therefore, that despite SN-38 loading differences between the micelle, equivalent or similar overall concentrations of SN-38 are being delivered to these tumors.

In summary, IT-141 is a novel SN-38-loaded polymer micelle with superior pharmacokinetics and antitumor activity compared to irinotecan. Although irinotecan is effective in the clinic, the ability to deliver SN-38 could be a superior treatment option for many patients. These data suggest that IT-141 may show activity in patients with solid tumors.

## 5. Conclusions

IT-141 is a micelle containing encapsulated SN-38 that was designed for systemic delivery. IT-141 increased the solubility of SN-38 by *∼*6,000-fold and had a diameter of 130 nm. IT-141 demonstrated superior pharmacokinetics to irinotecan and potent antitumor activity in HT-29 and HCT-116 colorectal cancer xenograft models. In summary, IT-141 is a promising new therapeutic agent for colorectal cancer that warrants clinical investigation.

## Figures and Tables

**Table 1:** IC50 values (*μ*M) of IT-141 compared to free SN-38 and irinotecan in cancer cell lines. Data are presented as mean ± standard deviation.

	Cell line	IT-141 (*μ*M)	Free SN-38 (*μ*M)	Irinotecan (*μ*M)
Colon	HT-29	0.0374 ± .001	0.0452 ± .002	24.6 ± 6.4
	HCT-116	0.165 ± .021	0.133 ± .037	16.3 ± 1.3
Prostate	PC-3	>5.0	>5.0	>100
	LNCaP	0.081 ± 0.006	0.083 ± 0.008	10.7 ± 2.2
Osteosarcoma	MG-63	0.080 ± 0.004	0.076 ± 0.002	7.2 ± 0.9
Pancreatic	BxPC-3	0.072 ± 0.004	0.072 ± 0.002	6.5 ± 1.3
Breast	MDA-MB-453	0.679 ± 0.006	0.661 ± 0.038	>100
	MDA-MB-231	>1.0	>1.0	66.3 ± 6.0
	MCF-7	0.717 ± 0.141	0.529 ± 0.017	86.0 ± 4.6
	BT-474	>3.0	>3.0	>200
Lung	A549	0.091 ± 0.002	0.099 ± 0.001	7.7 ± 1.0

**Table 2:** Plasma and tumor pharmacokinetics of IT-141 compared to irinotecan. Plasma AUC = *μ*g∗h/mL, tumor AUC = *μ*g∗h/g.

Drug	AUC	Half-life (h)	*C*_max⁡_ (*μ*g/mL)
IT-141 (plasma)	34.6	8.5	209.5
Irinotecan (plasma)	2.5	1.6	1.0
IT-141 (tumor)	16.4	3.9	9.4
Irinotecan (tumor)	1.9	17.5	0.2
